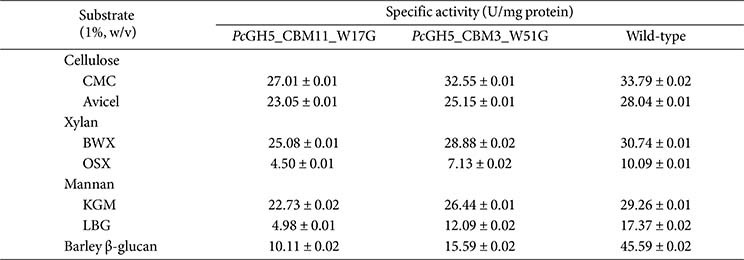# Role of the CBM11, Fn3, and CBM3 Domains in Enhancing the Multifunctional Enzymatic Activities of Glycoside Hydrolase Family 5 from *Paenibacillus curdlanolyticus* B-6

**DOI:** 10.4014/jmb.2507.07030

**Published:** 2025-10-29

**Authors:** Niendy Virnanda Fatmawati, Apinya Singkhala, Prattana Ketbot, Sirilak Baramee, Rattiya Waeonukul, Chakrit Tachaapaikoon, Ayaka Uke, Akihiko Kosugi, Khanok Ratanakhanokchai, Patthra Pason

**Affiliations:** 1 Division of Biochemical Technology, School of Bioresources and Technology, King Mongkut's University of Technology Thonburi, Bangkok 10150, Thailand; 2 Excellent Center of Enzyme Technology and Microbial Utilization, Pilot Plant Development and Training Institute, King Mongkut's University of Technology Thonburi, Bangkok 10150, Thailand; 3 Biological Resources and Post-harvest Division, Japan International Research Center for Agricultural Sciences, Tsukuba, Ibaraki 305-8686, Japan

**Keywords:** Carbohydrate-binding module, fibronectin type 3, glycoside hydrolase family 5, multifunctional enzyme, non-catalytic domain, *Paenibacillus curdlanolyticus*

## Abstract

Glycoside hydrolase family 5 (GH5) from *Paenibacillus curdlanolyticus* B-6 (*Pc*GH5) is a modular protein consisting of a GH5 catalytic domain and three non-catalytic domains (NCDs): a family 11 carbohydrate-binding module (CBM11), a fibronectin type 3 (Fn3), and a family 3 carbohydrate-binding module (CBM3). *Pc*GH5 exhibits unique properties compared with other GH5 members; it shows multifunctional endo-cellulase, endo-xylanase, endo-mannanase, and endo-1,3-1,4-β-glucanase activities. To understand the function of each NCD, recombinant full-length *Pc*GH5 and truncated variants were analyzed. Compared with the GH5 catalytic domain alone, stepwise addition of CBM11, Fn3, and CBM3 enhanced substrate binding and improved enzymatic activities towards polysaccharides, as confirmed by Fourier transform infrared spectroscopy (FTIR). Each NCD contributed distinctly: CBM11 bound 1,3-1,4-β-glucan, xylan and mannan with limited branching; Fn3 bound only cellulose; and CBM3 significantly improved binding to 1,3-1,4-β-glucan and highly branched xylan and mannan. There was a higher percentage of surface-exposed aromatic amino acids in CBM11 and CBM3 of *Pc*GH5, important for hydrophobic interactions with sugar rings, compared to other CBM11 and CBM3 members. Unlike other CBMs, sequence alignment and structural modelling revealed that *Pc*GH5 CBM11 and CBM3 have extra and/or more surface-exposed aromatic amino acids, which could interact with various oligosaccharide ligands such as hexose (cellotetraose and mannotetraose) and pentose (xylotetraose) via hydrophobic interactions, affecting enzyme activity. Mutagenesis confirmed Trp17 (CBM11) and Trp51 (CBM3) as key residues for insoluble substrates binding and enzymatic enhancement. Therefore, these aromatic amino acids are key factors improving substrates binding and enzymatic activities of *Pc*GH5 towards different β-1,4 glycosidic polysaccharide substrates.

## Introduction

The glycoside hydrolase family 5 (GH5) is one of the GH families; most of its members are endo-cellulases. However, some members in this family exhibit multifunctional activities, such as endo-cellulase/endo-mannanase/endo-1,3-1,4-β-glucanase in *Caldanaerobius polysaccharolyticus* [1] and endo-cellulase, endo-xylanase, endo-mannanase, and endo-1,3-1,4-β-glucanase in *Paenibacillus curdlanolyticus* strain B-6 [2]. These enzymes catalyse the hydrolysis of a wide range of β-glycosicis bonds, including cellulose, xylan, mannan, and 1,3-1,4-β-glucan, reflecting the broad specificity of the enzymes and their adaptability to catalyse a wide range of substrates. These enzymes are important for the degradation of insoluble polysaccharide substrates to oligosaccharides, which can be used as prebiotics in various food products [2, 3].

The members of GH families, including GH5, are modular proteins often associated with ancillary non-catalytic domains (NCDs) such as carbohydrate-binding modules (CBMs) and the fibronectin type-3 homology (Fn3) module [2, 4]. CBMs are ubiquitous in nature and present in enzymes produced by bacteria, fungi, and archaea [5]. According to amino acid sequence similarity and the three-dimensional (3D) structure, CBMs can be classified into several families. Specifically, as of 7 July 2025, of the 592,631 from different sources reported in the Carbohydrate Active enZYmes Database, CBMs can be divided into 106 families [http://www.cazy.org/cbm.html].

CBM11s have been reported to bind to a variety of polysaccharides such as cellulose, xylan, 1,3-1,4-β-glucan, and mannan [6]. Fn3s have been reported to bind to both soluble and insoluble cellulose substrates [7]. CBM3 can bind to polysaccharides such as cellulose, mannan, [8], xylan [9], cellulose, and 1,3-1,4-β-glucan [10, 11]. The function of CBMs is to bind to insoluble substrates and to support the enzymatic activity of the catalytic domain by increasing substrate proximity, resulting in improved catalytic efficiency [12]. On the other hand, the function of Fn3 remains unclear; however, it has been reported to act as a cellulose surface modifier to enhance the catalytic activity of cellulase towards insoluble and crystalline celluloses [13].

In previous work, we found that *P. curdlanolyticus* B-6 encodes a multifunctional GH5 (*Pc*GH5), which consists of a catalytic domain connected to three NCDs: CBM11, Fn3, and CBM3. These NCDs are important for promoting the multifunctional activities of *Pc*GH5 on the β-1,4 glycosidic linkages of crystalline cellulose, xylan, mannan, and 1,3-1,4-β-glucan, as well as complex polysaccharides contained in natural biomass [2]. In this study, we investigated the role of these three NCDs in enhancing the multifunctional activities of *Pc*GH5. Specifically, we cloned, expressed, purified, and characterised the full-length *Pc*GH5 enzyme (*Pc*GH5CD-CBM11-Fn3-CBM3) along with truncated variants containing one or more NCDs, namely *Pc*GH5CD-CBM11-Fn3 (containing the catalytic domain, CBM11, and Fn3), *Pc*GH5CD-CBM11 (containing the catalytic domain and CBM11), and *Pc*GH5CD (containing only the catalytic domain) in *Escherichia coli*. Thereafter, we examined the role of each NCD in *Pc*GH5.

## Materials and Methods

### Chemicals

Carboxymethyl cellulose (CMC; 400–800 cps), Avicel PH-101, glucose, birchwood xylan (BWX), oat spelt xylan (OSX), locust bean gum (LBG), barley 1,3-1,4-β-glucan, and bovine serum albumin were purchased from Sigma-Aldrich (USA). Konjac glucomannan (KGM), cello-oligosaccharides (G2–G6), manno-oligosaccharides (M2–M6), and xylo-oligosaccharides (X2–X6) were obtained from Megazyme (Ireland). Mannose and xylose were purchased from Merck (Germany). All other reagents and chemicals were of analytical grade.

### Organisms, Media, and Growth Conditions

Chromosomal DNA was extracted from *P. curdlanolyticus* B-6 cultured in Berg’s mineral salt medium at pH 7.0 [14]. The pET-28a(+) plasmid (Novagen, Germany) was used as the cloning vector, while *E. coli* DH5α and *E. coli* BL21 (DE3) (New England Biolabs, USA) were employed as the cloning and expression hosts, respectively. Transformants were selected by growing *E. coli* in Luria-Bertani (LB) medium supplemented with 50 μg/ml kanamycin at 37°C.

### Gene Cloning

*P. curdlanolyticus* B-6 is deposited in the BIOTEC Culture Collection at the National Center for Genetic Engineering and Biotechnology (BIOTEC), Thailand, under accession number BCC 11175. Chromosomal DNA was extracted from *P. curdlanolyticus* B-6 using the phenol-chloroform method. Based on the putative GH5 gene identified in the partial genome sequence of *P. curdlanolyticus* B-6, specific oligonucleotide primers were designed to amplify two truncated variants, *Pc*GH5CD-CBM11 and *Pc*GH5CD-CBM11-Fn3. Note that *Pc*GH5CD-CBM11-Fn3-CBM3 and *Pc*GH5CD have been studied previously [2].

For *Pc*GH5CD-CBM11 and *Pc*GH5CD-CBM11-Fn3, primers were designed to incorporate *Bam*HI-HF and *Not*I-HF restriction sites (indicated in bold). The forward primer for both constructs was 5'-GGGGG**GGATCC**G CTGAAGCTGTCGACTTAGAAAATCTG-3'. The reverse primers were 5'-TTTT**GCGGCCGC**TTACACTGT TCCTTCTGGCAATCC-3' for *Pc*GH5CD-CBM11 and 5'-TTTG**GCGGCCGC**TTACGATACTGCACTGCA AGATTAATTGG-3' for *Pc*GH5CD-CBM11-Fn3.

Polymerase chain reaction (PCR) amplification was performed using chromosomal DNA from *P. curdlanolyticus* B-6 as the template, following the manufacturer’s instructions with Ex Taq DNA polymerase (New England Biolabs, UK). The PCR products were digested with *Bam*HI-HF and *Not*I-HF (New England Biolabs), visualised by agarose gel electrophoresis, and purified using the Qiagen PCR purification kit (Qiagen, USA). The purified fragments were ligated into the corresponding sites of the pET-28a(+) vector to generate pET28a_*Pc*GH5CD-CBM11 and pET28a_*Pc*GH5CD-CBM11-Fn3. The recombinant plasmids used to transform *E. coli* DH5α, and positive colonies were screened via colony PCR and confirmed by DNA sequencing.

### Recombinant Protein Expression and Purification

The recombinant plasmids pET28a_*Pc*GH5CD-CBM11 and pET28a_*Pc*GH5CD-CBM11-Fn3 were used to transform *E. coli* BL21 (DE3), following the same procedure used for pET28a_*Pc*GH5CD-CBM11-Fn3-CBM3 and pET28a_*Pc*GH5CD as described previously [2]. Transformants were cultured in Luria-Bertani (LB) medium supplemented with kanamycin (50 μg/ml) at 37°C with shaking at 200 rpm until reaching an optical density at 600 nm (OD_600_) of 0.6. Protein expression was induced by adding 1 mM isopropyl β-D-1-thiogalactopyranoside, followed by incubation at 16°C for 18 h. After induction, cells were harvested, washed, and lysed by sonication. The lysates were collected by centrifugation at 8,000 rpm for 30 min at 4°C. Recombinant proteins were purified from the supernatant using a HisTrap HP affinity chromatography column (GE Healthcare, Japan). Protein purity was assessed by sodium dodecyl sulfate–polyacrylamide gel electrophoresis (SDS-PAGE) and cellulase zymogram analysis [2].

### Protein Determination and Enzymatic Activity Assays

Protein concentrations were determined using the Bradford assay [15], with bovine serum albumin serving as the standard. Enzymatic reactions were carried out in 50 mM sodium acetate buffer (pH 6.0) at 50°C, using 1 μM of purified enzyme and 1% (w/v) of each substrate. The release of reducing sugars from various substrates, including CMC, Avicel (PH-101), BWX, OSX, KGM, LBG, and barley 1,3-1,4-β-glucan, was measured using the Somogyi–Nelson method [16]. One unit (U) of enzyme activity was defined as the amount of enzyme required to release 1 μmol of reducing sugar per minute under assay conditions.

### Sequence-Based Amino Acid Analysis and Prediction of the Surface-Exposed Amino Acids

The amino acid sequences of the three NCDs (CBM11, Fn3, and CBM3) were submitted to the NCBI BLAST tool [https://blast.ncbi.nlm.nih.gov/Blast.cgi] to identify homologous proteins with high sequence identity. The surface-exposed amino acid residues were predicted using NetSurfP-3.0 [https://services.healthtech.dtu.dk/services/NetSurfP-3.0/, accessed on 7 July 2025] [17].

### Ability of Enzyme to Bind to Polysaccharide

Polysaccharide binding was assessed by incubating full-length *Pc*GH5 (*Pc*GH5CD-CBM11-Fn3-CBM3), *Pc*GH5CD-CBM11-Fn3, *Pc*GH5CD-CBM11, and *Pc*GH5CD with various substrates. Reaction mixtures were prepared containing 90 μl (1%, w/v) suspensions of each polysaccharide (Avicel PH-101, BWX, OSX, KGM, LBG, and barley 1,3-1,4-β-glucan) and 10 μl of each purified enzyme (1 μM). As a control, 90 μl of distilled water was mixed with 10 μl of each purified enzyme. All mixtures were incubated at 4°C with occasional stirring for 30 min, followed by centrifugation at 10,000 rpm for 5 min. The supernatants were collected and analysed by SDS-PAGE. A visible protein band in the control lane indicated unbound protein, while reduced or absent bands in the substrate lanes indicated protein binding to the polysaccharide.

### 3D Structural Modelling

3D structural models of *Pc*GH5 CBM11, Fn3, and CBM3 were generated based on homology with proteins identified in the Protein Data Bank (PDB) [http://www.rcsb.org/pdb/home/home.do]. Ligands relevant to each domain were retrieved from PubChem [https://pubchem.ncbi.nlm.nih.gov/] and used for molecular docking analysis with PyRx [18]. Structural visualisation and domain–ligand interaction analysis were carried out using PyMOL [https://pymol.org/], providing detailed insights into the molecular interactions of each domain.

### FTIR Spectroscopy Analysis

The functional groups of untreated and enzyme-treated Avicel were analyzed using FTIR spectroscopy. Avicel samples were hydrolyzed with 1 μM of the full-length *Pc*GH5 enzyme and its truncated enzymes. Spectra were recorded on a PerkinElmer FTIR spectrometer (Midland, ON, Canada) equipped with a universal attenuated total reflectance accessory with an internal diamond crystal. Measurements were performed in transmission mode in the wavenumber range of 500–4,000 cm^-1^. All spectra were collected at room temperature, and baseline corrections were applied prior to analysis.

### Mutagenesis of Surface-Exposed Aromatic Residues

The pET-28a vector carrying the *Pc*GH5 gene encoding wild-type *Pc*GH5CD-CBM11-Fn3-CBM3 (full-length) was used as the template. To investigate the role of surface-exposed aromatic residues in the NCDs, two mutants were designed: W17G in CBM11 and W51G in CBM3. Both mutants were synthesized *de novo* by U2Bio (Bangkok, Thailand), with codon optimization for expression in *E. coli* BL21. For W17G, the wild-type TGG codon encoding tryptophan at nucleotide positions 1033-1035 in CBM11 was replaced with GGG encoding glycine. For W51G, the wild-type TGG codon at nucleotide positions 2083-2085 in CBM3 encoding tryptophan was replaced with GGG encoding glycine. The synthesized genes were provided in a cloning-ready format within the pET-28a vector backbone for subsequent cloning and protein expression.

### Degradation Product Analysis

The enzymatic activities of full-length *Pc*GH5 (*Pc*GH5CD-CBM11-Fn3-CBM3), *Pc*GH5CD-CBM11-Fn3, *Pc*GH5CD-CBM11, and *Pc*GH5CD were evaluated using 1% (w/v) CMC, Avicel (PH-101), BWX, OSX, KGM, LBG, and barley 1,3-1,4-β-glucan as substrates. Each reaction was conducted in a 500-μl mixture containing 1 μM of the purified enzyme in sodium acetate buffer (pH 6.0) and incubated at 50°C for 30 min. The resulting hydrolysis products were analysed by thin-layer chromatography (TLC) on silica gel 60 F254 plates (Merck) using a solvent system of acetic acid:n-butanol:water (1:2:1, v/v/v). Sugar spots were visualised by spraying the plates with a detection reagent composed of 4 g of α-diphenylamine, 4 ml of aniline, 200 ml of acetone, and 30 ml of 80%(v/v) phosphoric acid, followed by heating at 100°C [19].

## Results and Discussion

### Modular Structure and Truncation Strategy of *Pc*GH5

**Fig. 1:**
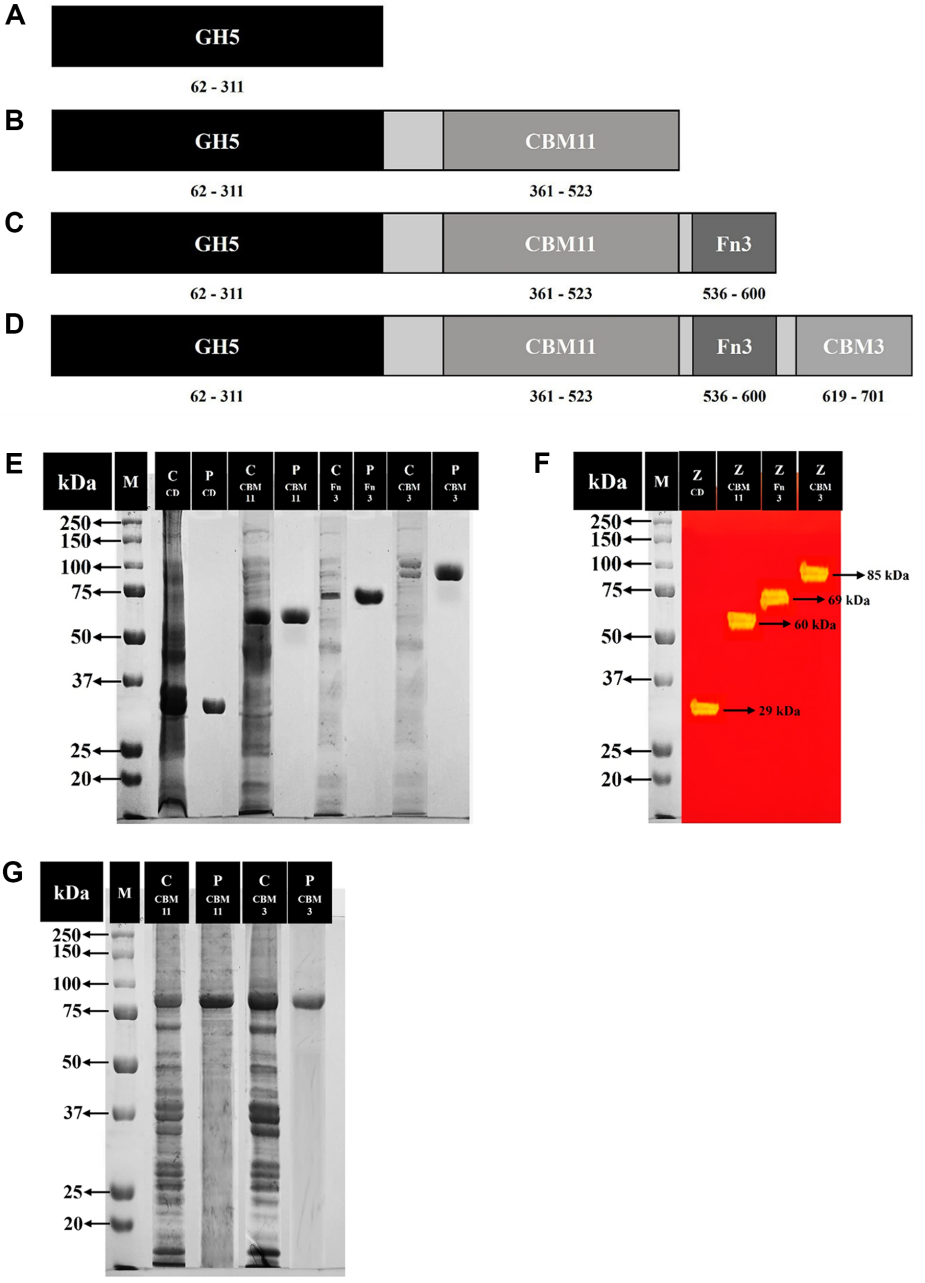
Schematic illustration of the modular structures of the GH5 catalytic domain (*Pc*GH5CD) (only the catalytic domain) (**A**), the truncated *Pc*GH5CD-CBM11 (the catalytic domain and family 11 carbohydrate-binding module) (**B**), the truncated *Pc*GH5CD-CBM11-Fn3 (the catalytic domain, family 11 carbohydrate-binding module, and fibronectin type 3 domain) (**C**), and the full-length *Pc*GH5CD-CBM11-Fn3-CBM3 (the catalytic domain, family 11 carbohydrate-binding module, fibronectin type 3 domain, and family 3 carbohydrate-binding module) (**D**) from *Paenibacillus curdlanolyticus* B-6. Sodium dodecyl sulfate–polyacrylamide gel electrophoresis (SDS-PAGE) analysis of purified recombinant proteins (**E**). Lane M, protein marker; lane C, crude enzyme; lane P, purified enzyme; lane CD, *Pc*GH5CD; lane CBM11, *Pc*GH5CD-CBM11; lane Fn3, *Pc*GH5CD-CBM11-Fn3; lane CBM3, *Pc*GH5CD-CBM11-Fn3-CBM3. Cellulase zymogram analysis of purified recombinant proteins (**F**). Lane M, protein marker; lane Z, purified enzyme; *Pc*GH5CD (CD) (29 kDa), *Pc*GH5CD-CBM11 (CBM11) (60 kDa), *Pc*GH5CD-CBM11-Fn3 (Fn3) (69 kDa), and *Pc*GH5CD-CBM11-Fn3-CBM3 (CBM3) (85 kDa). SDS-PAGE analysis of purified recombinant mutant proteins (**G**). Lane M, protein marker; lane C CBM11, crude mutant *Pc*GH5_CBM11_W17G enzyme; lane P CBM11, purified mutant *Pc*GH5_CBM11_W17G enzyme; lane C CBM3, crude mutant *Pc*GH5_CBM3_W51G enzyme; lane P CBM3, purified mutant *Pc*GH5_CBM3_W51G enzyme.

Based on the partial genome analysis of *P. curdlanolyticus* B-6, we identified the *Pc*GH5 gene, which encodes a GH5 catalytic domain and three NCDs: CBM11, Fn3, and CBM3. The *Pc*GH5 catalytic domain is located at residues 62–311, followed by CBM11 (residues 361–523), Fn3 (residues 536–600), and CBM3 (residues 619–701)(Fig. 1A–1D). To elucidate the function of these NCDs, we generated full-length *Pc*GH5 as well as three truncated forms of the enzyme. *Pc*GH5CD retained only the catalytic domain, with all NCDs were removed (Fig. 1A); it served as a reference to determine the role of each NCD. *Pc*GH5CD-CBM11 included the GH5 catalytic domain and CBM11 (Fig. 1B); it allowed us to assess the contribution of CBM11 to substrate binding and the enhancement of enzyme activity towards insoluble substrates compared with *Pc*GH5CD. *Pc*GH5CD-CBM11-Fn3 comprised the GH5 catalytic domain, followed by CBM11 and Fn3 (Fig. 1C); we used it to investigate the functions of Fn3 compared with *Pc*GH5CD-CBM11. Finally, *Pc*GH5CD-CBM11-Fn3-CBM3 included the GH5 catalytic domain and all three NCDs (Fig. 1D); we employed it to examine the functions of CBM3 compared with *Pc*GH5CD-CBM11-Fn3.

### Expression and Purification of the Recombinant Enzymes

We ligated four genes – namely *pcgh5CD*, *pcgh5CD-CBM11*, *pcgh5CD-CBM11-Fn3*, and *pcgh5CD-CBM11-Fn3-CBM3* (full sequence) into the pET-28a vector, yielding pET28*Pc*GH5CD, pET28*Pc*GH5CD-CBM11, pET28*Pc*GH5CD-CBM11-Fn3, and pET28*Pc*GH5CD-CBM11-Fn3-CBM3, respectively, which we introduced into *E. coli* DH5α. Each recombinant gene was successfully expressed in *E. coli* BL21 (DE3). Each protein contained an N-terminal histidine tag and was purified using a HisTrap HP column. After purification, *Pc*GH5CD, *Pc*GH5CD-CBM11, *Pc*GH5CD-CBM11-Fn3, and *Pc*GH5CD-CBM11-Fn3-CBM3 showed a single band on SDS-PAGE (Fig. 1E) and cellulase zymogram (Fig. 1F), with a molecular mass of approximately 29, 60, 69, and 85 kDa, respectively, consistent with the calculated values. We used these purified proteins for the subsequent biochemical experiments.

### Amino Acids Sequence Analysis of CBM11, Fn3, and CBM3

**Fig. 2:**
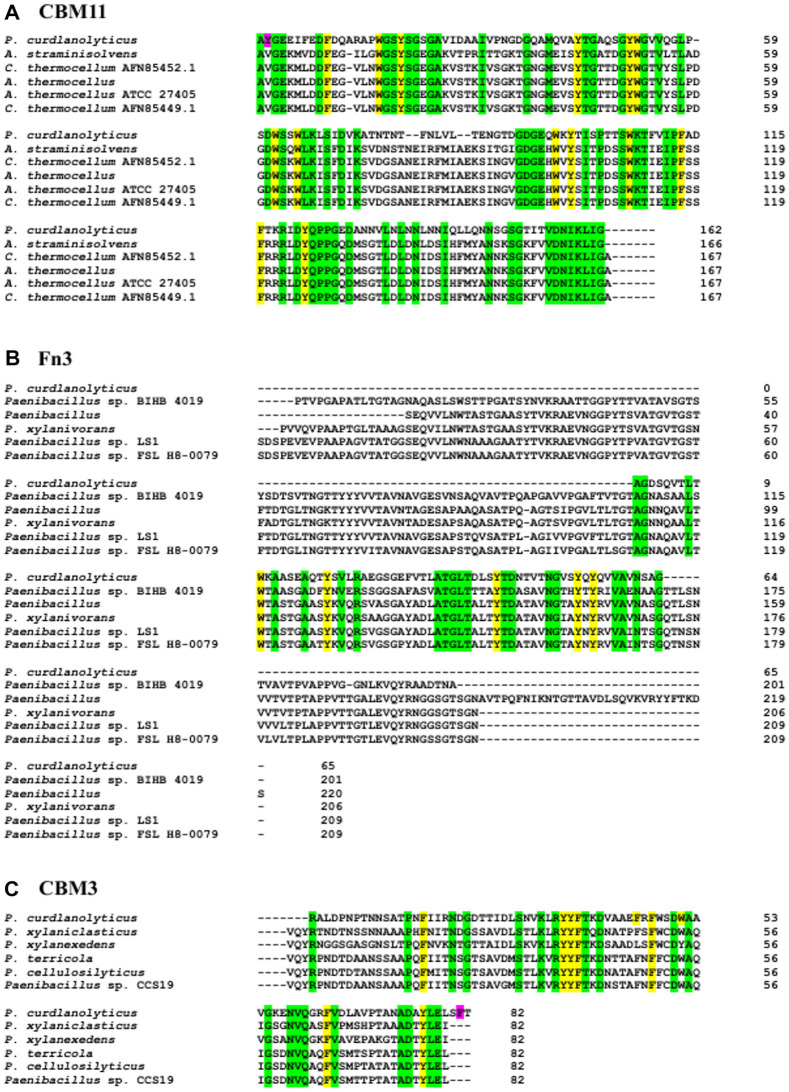
(**A**) Amino acid sequence alignment of the CBM11 non-catalytic domain from *P. curdlanolyticus* strain B-6 (this work) compared with other characterised CBM11 from *Acetivibrio straminisolvens* (WP_265442738.1), *Clostridium thermocellum* (AFN85452.1), *A. thermocellus* (WP_011838089.1), *A. thermocellus* ATCC 27405 (2LRO_A), and *C. thermocellum* (AFN85449.1). (**B**) Amino acid sequence alignment of the Fn3 non-catalytic domain from *P. curdlanolyticus* strain B-6 (this work) compared with other characterised Fn3 from *Paenibacillus* sp. BIHB 4019 (WP_099517176.1), *Paenibacillus* sp. (WP_109997962.1), *P. xylanivorans* (WP_053783364.1), *Paenibacillus* sp. LS1 (WP_264931671.1), and *Paenibacillus* sp. FSL H8-0079 (WP_340400803.1). (**C**) Amino acid sequence alignment of the CBM3 non-catalytic domain from *P. curdlanolyticus* strain B-6 (this work) compared with other characterised CBM3 from *P. xylaniclasticus* (WP_246028019.1), *P. xylanexedens* (WP_145411939.1), *P. terricola* (WP_318152810.1), *P. cellulosilyticus* (WP_281272002.1), and *Paenibacillus* sp. CCS19 (WP_317968269.1). The conserved aromatic amino acid residues are highlighted in yellow, the conserved non-aromatic amino acid residues are highlighted in green, and the extra surface-exposed aromatic amino acid residues in *Pc*GH5 CBM11 or *Pc*GH5 CBM3 are highlighted in pink (these residues are not present in the other CBM11 and CBM3 members).

To investigate the similarities and potential functions of the amino acids in the *Pc*GH5 NCDs, we performed multiple sequence alignment of each NCD. With this approach, we compared the amino acid sequence of the CBM11, Fn3, and CBM3 domains in *Pc*GH5 with other characterised proteins to identify residues potentially involved in substrate binding. BlastP analysis of *Pc*GH5 CBM11 revealed a sequence identity ranging from 25% to 47% when aligned with other characterised CBM11 members. We observed the highest sequence similarity with CBM11 from *Acetivibrio straminisolvens* (WP_265442738.1, 47.02%), followed by *Clostridium thermocellum* (AFN85452.1, 46.71%), *A. thermocellus* (WP_011838089.1, 46.71%), *A. thermocellus* ATCC 27405 (2LRO_A, 46.47%), and *C. thermocellum* (AFN85449.1, 46.2%) (Fig. 2A). Compared with other characterised CBM11 members, we found several conserved amino acids (highlighted in yellow for aromatic amino acid residues and in green for non-aromatic amino acid residues); they may play some functional role in CBM11.

BlastP analysis of *Pc*GH5 Fn3 revealed a sequence identity ranging from 48% to 50% compared with other characterised Fn3 members. The highest sequence similarity was with the Fn3 domain from *Paenibacillus* sp. BIHB 4019 (WP_099517176.1, 50.00%), followed by *Paenibacillus* sp. (WP_109997962.1, 49.62%), *P. xylanivorans* (WP_053783364.1, 49.62%), *Paenibacillus* sp. LS1 (WP_264931671.1, 48.85%), and *Paenibacillus* sp. FSL H8-0079 (WP_340400803.1, 48.85%) (Fig. 2B). Compared with other characterised Fn3 members, we found several conserved amino acids (highlighted in yellow for aromatic amino acid residues and in green for non-aromatic amino acid residues). They may play a functional role in Fn3.

According to the CAZy database [http://www.cazy.org/CBM3.html], up to 22 3D structures of CBM3 have been reported, indicating that this type of CBM is of great interest for studying the binding and hydrolysis of β-1,4-glycosidic polysaccharide substrates. BlastP analysis of *Pc*GH5 CBM3 showed a sequence identity ranging from 46% to 60% compared with other characterised CBM3 members. We noted the highest sequence similarity with CBM3 from *P. xylaniclasticus* (WP_246028019.1, 59.77%), followed by *P. xylanexedens* (WP_145411939.1, 56.32%), *P. terricola* (WP_318152810.1, 54.65%), *P. cellulosilyticus* (WP_281272002.1, 53.49%), and *Paenibacillus* sp. CCS19 (WP_317968269.1, 51.14%) (Fig. 2C). Compared with other characterised CBM3 members, there were several conserved amino acids (highlighted in yellow for aromatic amino acid residues and in green for non-aromatic amino acid residues) that may play a functional role in CBM3.

### Number and Percentage of Aromatic Amino Acid on the Surface of CBM11, Fn3, and CBM3

It is well known that aromatic amino acids in GHs interact with cyclic rings of various sugar chains, including hexose (glucose, mannose) and pentose (xylose) sugars of oligosaccharide/polysaccharide substrates, by hydrophobic interactions via stacking and/or sandwich interactions [20]. We analysed the predicted exposed and buried amino acid residues of the *Pc*GH5 NCDs – CBM11, Fn3, and CBM3 – by using the NetSurfP-3.0 [https://services.healthtech.dtu.dk/services/NetSurfP-3.0/, accessed on 7 July 2025] (Fig. S1). In addition, we calculated the percentage of aromatic amino acids on the protein surface of CBM11, Fn3, and CBM3 from *Pc*GH5 and CBM11s, Fn3s, and CBM3s from other microbes based on the predictions obtained using NetSurfP-3.0. Table 1 summarises the surface-exposed aromatic amino acids, total number of amino acids, and percentage of surface-exposed aromatic amino acids in the protein of each *Pc*GH5 NCD with the amino acids of other microbial NCD members.

Compared with other CBM11 members, *Pc*GH5 CBM11 exhibited a higher number of surface-exposed aromatic amino acids. Four of the 162 total amino acids (2.46%) in *Pc*GH5 CBM11 are surface-exposed aromatic amino acids. Among the other CBM11 members, the highest number of surface-exposed aromatic amino acids is 2 out of 166 total amino acids (1.20%). Of the four surface-exposed aromatic amino acids (Tyr2, Trp17, Tyr20, and Tyr122) in *Pc*GH5 CBM11, Tyr2 is absent from the other CBM11s (Table 1), where only Trp17 from *Pc*GH5 is exposed (Fig. S2). These two extra surface-exposed aromatic amino acid residues may cause *Pc*GH5 to function differently from other CBM11s. Ichikawa *et al*. [6] reported that surface-exposed aromatic amino acids are important for interaction with polysaccharide substrates and promote enzymatic activity. Thus, an increase in the number of surface-exposed aromatic amino acid residues in *Pc*GH5 CBM11 may increase the binding of sugar rings of oligosaccharide and polysaccharide substrates via hydrophobic interactions. This improved binding of *Pc*GH5 to polysaccharide substrates relative to other CBM11 members would increase enzymatic activities.

Surprisingly, *Pc*GH5 CBM3 has more surface-exposed aromatic amino acids (8 out of 82 residues, or 9.75%) than the other CBM3s (a maximum 2 out of 82 residues, or 2.43%) (Table 1). These eight aromatic amino acids (Phe16, Tyr35, Tyr36, Phe37, Phe45, Phe47, Trp51, and Phe81) may interact with the sugar cyclic rings by hydrophobic interactions via stacking and/or sandwich interactions [20], which are expected to facilitate the binding of *Pc*GH5 to various β-1,4-linked polysaccharide substrate, including cellulose, xylan, mannan, and 1,3-1,4-β-glucan. On the other hand, the scaffolding *Cj*CBM3 from *Clostridium josui* binds to cellulose and mannan [8], CtCBM3-0271 from *C. thermocellum* binds to xylan [9], CBM3 of GH9 endo-cellulase from *Hungateiclostridium thermocellum* ATCC 27405 binds to cellulose and 1,3-1,4-β-glucan [10], and CBM3 from *Bacillus* sp. KD1014 binds to 1,3-1,4-β-glucan [11].

In contrast, *Pc*GH5 Fn3, and other Fn3 members show a low abundance of surface-exposed aromatic amino acids (from 0 of 209 residues to 1 of 209 residues) (Table 1). Jee *et al*. [21] reported that there are almost no surface-exposed aromatic amino acid residues in Fn3, indicating that Fn3 plays a different role compared with other CBMs that require surface-exposed aromatic amino acid residues to function. These results suggest that the surface-exposed aromatic amino acid (Phe30) in *Pc*GH5 Fn3 may play a limited role in substrate binding and enhancing *Pc*GH5 enzymatic activity.

### Protein–Polysaccharide Binding

We performed protein–polysaccharide binding assays to gain insights into the polysaccharide substrate specificity of full-length *Pc*GH5 and the three truncated forms. After binding, we used SDS-PAGE to indicate the extent to which the proteins bind to the substrate. A reduction in bands in the supernatant indicates binding of the protein to the substrate, and the protein is pulled down along with the insoluble substrate. In contrast, strong bands indicate weak or no binding of the protein to the polysaccharides.

**Fig. 3:**
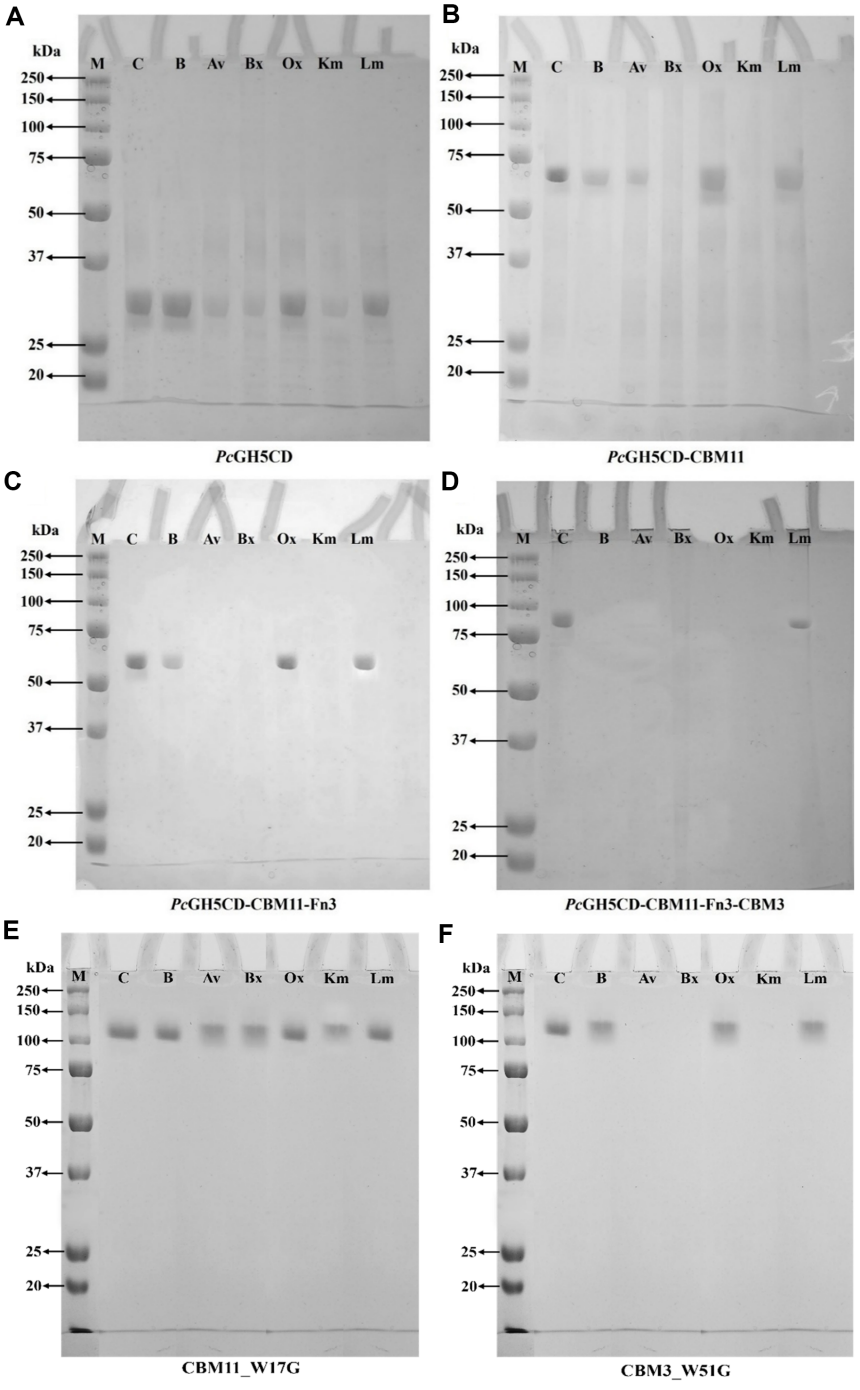
SDS-PAGE analysis of protein–polysaccharide binding. The presence of a protein band indicates weak or no binding to the polysaccharide, while the absence of a band indicates complete binding of the protein to the insoluble polysaccharide. Lane M, protein marker; lane C, control (no polysaccharide added); lane B, barley 1,3-1,4-β-glucan; lane Av, Avicel; lane Bx, birchwood xylan; lane Ox, oat spelt xylan; lane Km, Konjac glucomannan; lane Lm, locust bean gum. Representative results are shown for *Pc*GH5CD (**A**), *Pc*GH5CD-CBM11 (**B**), *Pc*GH5CD-CBM11-Fn3 (**C**), full-length *Pc*GH5CD-CBM11-Fn3-CBM3 (**D**), mutant *Pc*GH5_CBM11_W17G (**E**), and mutant *Pc*GH5_CBM3_W51G (**F**).

As shown in Fig. 3A, compared with the control (without any form of polysaccharide added), *Pc*GH5CD showed significant binding to Avicel (crystalline cellulose), BWX (low-branched xylan), and KGM (low-branched mannan). However, *Pc*GH5CD presented a low ability to bind to highly branched substrates such as LBG and OSX, and 1,3-1,4-β-glucan. We did not use CMC because it is a soluble substrate.

*Pc*GH5CD-CBM11 showed reduced band intensities in all lanes, especially with barley 1,3-1,4-β-glucan, while there were no detectable protein bands in the BWX and KGM lanes (Fig. 3B), indicating a significantly improved binding affinity compared with the catalytic domain alone (*Pc*GH5CD). This is consistent with the known role of CBM11 in enhancing the interaction between the catalytic domain and substrate. These results are consistent with previous reports highlighting the cooperative function of NCDs and catalytic domains in GHs [12, 22].

Compared with *Pc*GH5CD-CBM11, *Pc*GH5CD-CBM11-Fn3 showed significant binding to Avicel, with no protein band in that lane (Fig. 3C), suggesting Fn3 plays a role in binding to Avicel. The Fn3 domain from cellobiohydrolase CbhA of *C. thermocellum* has been reported to act as a cellulose fibre surface modifier that enhances cellulase activity towards insoluble cellulose [13], supporting its potential contribution when integrated into the GH5 catalytic architecture.

Fig. 3D shows that full-length *Pc*GH5 bound completely to all polysaccharides except for LBG, for which we could still detect a faint protein band. These results suggest that when the number of NCDs attached to the *Pc*GH5 catalytic domain increases, it significantly enhances substrate binding, which should promote the efficient binding of the catalytic domain to various polysaccharides. The observed binding patterns are consistent with specific enzyme activities (Table 2): the enzymes with more NCDs attached to the catalytic domain exhibited stronger polysaccharide binding and higher hydrolytic efficiency.

Among the variant construct enzymes, *Pc*GH5CD showed the weakest binding, as denoted by the protein bands in all polysaccharide lanes, with greater intensity and thickness (Fig. 3A). These results indicate that the catalytic domain alone has minimal binding to the tested polysaccharides, underscoring the importance of the NCDs in substrate recognition and binding. In the full-length enzyme, the three *Pc*GH5 NCDs might interact synergistically to bind to polysaccharides, but we cannot definitively draw this conclusion based on these data.

### Docking and Structural Modelling of NCDs with G4, X4, and M4

To confirm the functions of CBM11, Fn3, and CBM3 in *Pc*GH5 and to gain a more in-depth understanding of their substrate-binding properties, we used SWISS-MODEL for structural modelling. We used the crystal structures of CtCBM11 from *C. thermocellum* ATCC 27405 (PDB ID: 2LRO_A) [23] as a reference template since among the CBM11 members, 2LRO_A shows the highest sequence similarity to *Pc*GH5 CBM11, and its 3D structure and binding subsites have been described. Similarly, the Fn3 domain of the cellulosomal cellulase from *C. thermocellum* (PDB ID: 3MPC) [24], and CBM3 from the scaffolding protein CipA of *C. thermocellum* (PDB ID: 4B9F) [https://www.rcsb.org/structure/4B9F] were used as reference templates for *Pc*GH5 Fn3 and *Pc*GH5 CBM3, respectively.

*Pc*GH5 exhibits multifunctional enzyme activities of endo-cellulase, endo-xylanase, endo-mannanase, and endo-1,3-1,4-β-glucanase. Therefore, the individual functions of CBM11, Fn3 and CBM3 in *Pc*GH5 binding to various polysaccharides that may promote the binding and activity of the *Pc*GH5 catalytic domain were studied by structural modeling.

**Fig. 4:**
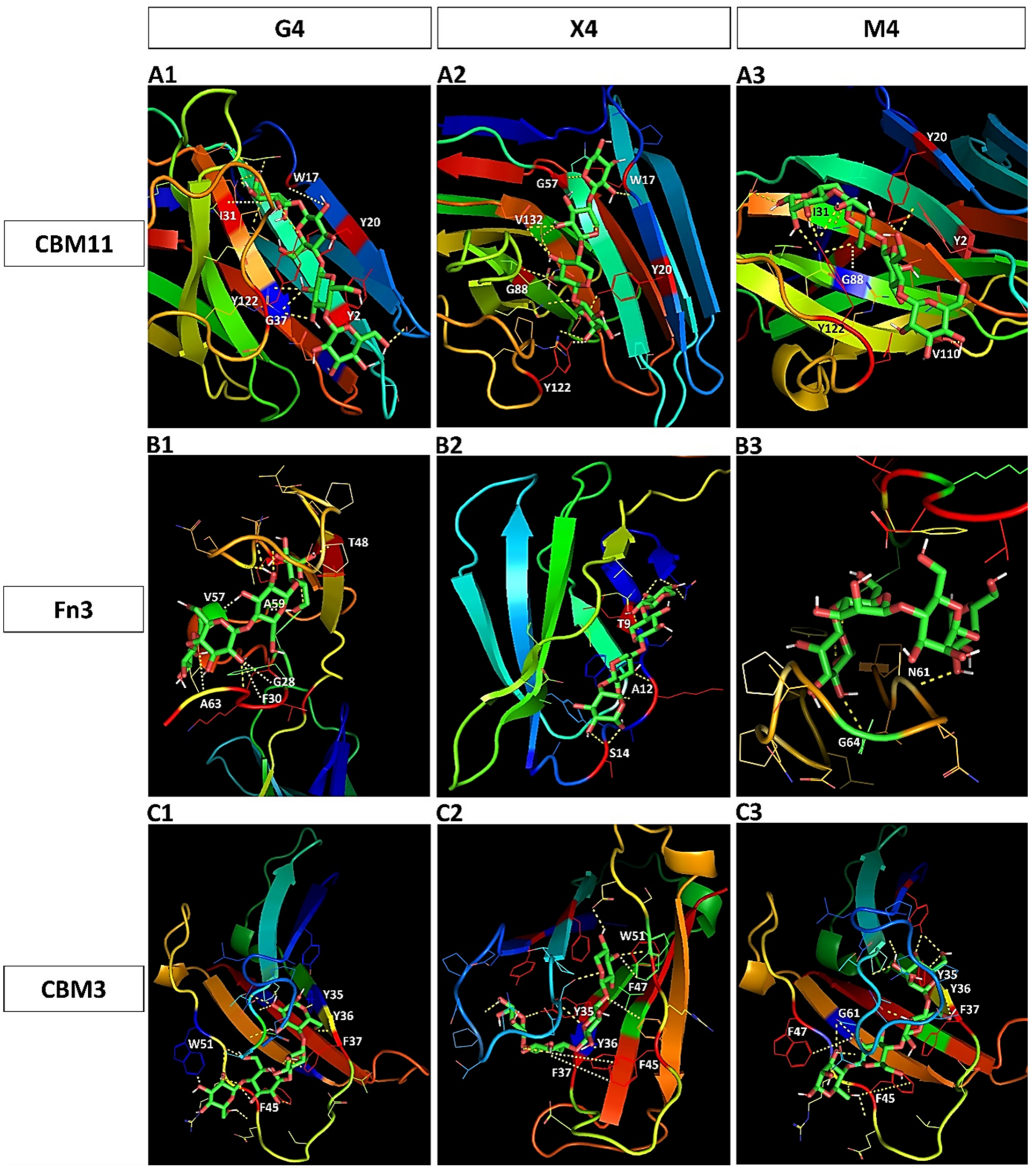
Structural modelling of three non-catalytic domains; CBM11 (A1–A3), Fn3 (B1–B3), and CBM3 (C1– C3), bound to cellotetraose (G4), xylotetraose (X4), and mannotetraose (M4), respectively. The predicted three-dimensional (3D) structures highlight amino acid residues at binding subsites expected to bind to cellulose (**A1, B1, C1**), xylan (**A2, B2, C2**), and mannan (**A3, B3, C3**). G4, X4, and M4 represent the sugar ligands for cellulose, xylan, and mannan that interact with CBM11, Fn3, and CBM3, respectively. The 3D structures were modelled using SWISS-MODEL [https://swissmodel.expasy.org/]. Ligands were collected from PubChem [https://pubchem.ncbi.nlm.nih.gov/], and ligand– macromolecule interactions were predicted using PyRx [Dallakyan and Olson, 2015]. Structural visualisation and analysis were performed with PyMOL [https://pymol.org/].

Fig. 4A shows the predicted binding subsites of *Pc*GH5 CBM11 on cellotetraose (G4), xylotetraose (X4), and mannotetraose (M4), respectively. Fig. 4A1 shows six amino acid residues – Tyr2 (Glc1); Gly37 (Glc2); Tyr20 and Tyr17 (Glc3); and Ile31 and Trp122 (Glc4) – located within the *Pc*GH5 CBM11 binding subsites, which bind to each of the G4 ligands. Fig. 4A2 shows six residues – Tyr20 and Tyr122 (Xyl1); Gly88 (Xyl2); Val132 (Xyl3); and Trp17 and Gly57 (Xyl4) – which are located within the binding subsites of each X4 ligand molecule. Six residues –Val110 (Man1); Tyr2 and Tyr20 (Man2); Ile31 and Gly88 (Man3); and Tyr122 (Man4) – bind to each molecule of the M4 ligand (Fig. 4A3). These results demonstrate that because the molecular structures of glucose, xylose and mannose differ, when the ligand changes, the amino acids present in the binding subsites move to the most suitable sugar molecule to interact.

Tables 1 and 3 and Figs. 2A and 4A show that, compared with other CBM11s, among the four surface-exposed aromatic amino acid residues (Tyr2, Trp17, Tyr20, Tyr122) in *Pc*GH5 CBM11, Tyr2 is an extra surface-exposed aromatic amino acid residue that binds to Glc1 of G4 ligand and Man2 of M4 ligand, while Trp17 binds to Glc3 of G4 ligand and Xyl4 of X4 ligand. In contrast, this aromatic amino acid residue is not exposed in other CBM11 members (Fig. S2). Therefore, after binding to the sugar rings, Tyr2 and Trp17 in CBM11 may facilitate *Pc*GH5 CBM11 binding to the sugar cyclic rings of various oligosaccharide and polysaccharide substrates and support the multifunctional enzymatic activities of the *Pc*GH5 catalytic domain in a way that other CBM11 members cannot.

Fig. 4B shows the predicted binding subsites of *Pc*GH5 Fn3 relative to different substrates. Fig. 4B1 shows six residues – Ala63 (Glc1); Gly28 and Phe30 (Glc2); Val57 and Ala59 (Glc3); and Thr48 (Glc4) – that are important for binding of each G4 ligand. Fig. 4B2 shows three residues – Ser14 (Xyl1); Ala12 (Xyl2); and Thr9 (Xyl4) – that bind to the X4 ligand. Fig. 4B3 highlights two residues, Gly46 (Man1) and Asn61 (Man4), that bind to the M4 ligand. On the other hand, Fig. 4C shows the binding subsites of *Pc*GH5 CBM3 on different substrates. As shown in Fig. 4C1, five residues – Trp51 (Glc1); Phe45 (Glc2); Phe37 (Glc3); and Tyr35, Tyr36 (Glc4) – bind to each G4 ligand. Fig. 4C2 shows six residues – Tyr35 (Xyl1); Phe37 and Phe45 (Xyl2); Tyr36 (Xyl3); and Phe47 and Trp51 (Xyl4) – bind to the X4 ligand. Fig. 4C3 highlights six residues – Phe45 (Man1); Phe47 and Gly61 (Man2); Tyr36 and Phe37 (Man3); and Tyr35 (Man4) – that bind to the M4 ligand.

Although eight aromatic amino acid residues (Phe16, Tyr35, Tyr36, Phe37, Phe45, Phe47, Trp51, and Phe81) are exposed on the surface of *Pc*GH5 CBM3 (Table 1), only six aromatic amino acid residues (Tyr35, Tyr36, Phe37, Phe45, Phe47, and Trp51) could interact with the G4, X4, and M4 ligands (Table 3 and Fig. 4). On the other hand, there are seven aromatic amino acid residues (Phe16, Tyr35, Tyr36, Phe37, Phe45, Phe47, and Trp51) that are conserved among other CBM3 members (Fig. 2); these residues are not present on the surface of those proteins (Fig. S3). As a result, only *Pc*GH5 CBM3 could bind to various polysaccharide sugar rings, such as cellulose, xylan, and mannan, and enhance the multifunctional enzymatic activities of the *Pc*GH5 catalytic domain.

Table 3 presents the distance (Å) between the amino acid residues in the binding subsites of CBM11, Fn3, and CBM3 interacting with G4, X4, and M4. The results indicate that all three NCDs have unique properties in the orientation to bind to each ligand. After binding, these NCDs likely bring the catalytic domain closer to the substrate and ultimately increase the enzymatic activity [12].

### Substrate Specificity of Full-length and Truncated *Pc*GH5 Enzymes on Polysaccharides

We studied the contribution of each NCD (CBM11, Fn3, and CBM3) in full-length *Pc*GH5 to the hydrolysis of various polysaccharides. As shown in Table 2, *Pc*GH5CD presented multifunctional enzymatic activities towards cellulose, xylan, mannan, and 1,3-1,4-β-glucan. Of note, *Pc*GH5CD showed better hydrolytic activity for lowbranched substrates (BWX, 16.21 U mg^-1^ protein, and KGM, 18.33 U mg^-1^ protein) than highly branched substrates (OSX, 3.33 U mg^-1^ protein, and LBG, 3.34 U mg^-1^ protein) and barley 1,3-1,4-β-glucan (4.54 U mg^-1^ protein). It also showed lower activity on crystalline cellulose (Avicel, 15.58 U mg^-1^ protein) compared with CMC (26.80 U mg^-1^ protein).

Compared with *Pc*GH5CD, *Pc*GH5CD-CBM11 showed similar activity towards CMC (26.98 U mg^-1^ protein vs 26.80 U mg^-1^ protein for *Pc*GH5CD), and a 1.25-fold increase in activity towards Avicel (19.46 U mg^-1^ protein) (Table 2). For xylan substrates, *Pc*GH5CD-CBM11 showed a 1.55-fold increase in activity towards BWX (25.07 U mg^-1^ protein) and a 1.91-fold increase in activity towards OSX (6.36 U mg^-1^ protein). For mannan substrates, it showed a 1.22-fold increase in activity towards KGM (22.32 U mg^-1^ protein) and a 2.97-fold increase in activity towards LBG (9.90 U mg^-1^ protein). In addition, the activity of *Pc*GH5CD-CBM11 towards barley 1,3-1,4-β-glucan (14.93 U mg^-1^ protein) was increased 3.29-fold.

We found that *Pc*GH5 CBM11 significantly improved the Avicelase, xylanase, and mannanase activities (Table 2). The CBM11 domain facilitate binding to insoluble substrates, thus improving the catalytic efficiency of GHs. Improving the interaction between enzyme and substrate is particularly beneficial for the degradation of insoluble substrates [12, 22]. Crystal structure and site-directed mutagenesis studies have revealed that aromatic amino acid residues in CBM11 contribute to interactions with polysaccharides via hydrophobic interactions, which play a key role in substrate specificity and enzymatic activity [6, 25]. The presence of CBM11 from *C. thermocellum* endo-cellulase H increases the affinity and enzymatic activity of *Dictyoglomus turgidum* endocellulase towards insoluble cellulose [26], while CBM11 from *Ruminoclostridium thermocellum* celH enhances the hydrolytic efficiency of 1,3-1,4-β-glucanase from *Bacillus subtilis* by up to 30% when hydrolysing 1,3-1,4-β-glucan from barley [27]. Similarly, CBM11 of a bifunctional endo-cellulase/endo-xylanase Lic26A-Cel5E from *C. thermocellum*, can interact with cellulose, xylan, 1,3-1,4-β-glucan, and mannan [25]. Thus, CBM11s are important for binding to insoluble polysaccharides and enhancing the activity of the catalytic domain on insoluble substrates.

We compared the activity of *Pc*GH5CD-CBM11-Fn3 to that of *Pc*GH5CD-CBM11. Table 2 shows that for cellulose substrates, *Pc*GH5CD-CBM11-Fn3 showed a 1.12-fold increase in activity towards CMC (30.31 U mg^-1^ protein), and 1.19-fold increased activity towards Avicel (23.11 U mg^-1^ protein). For xylan substrates, *Pc*GH5CDCBM11- Fn3 did not show a change in activity towards BWX (25.11 U mg^-1^ protein) and OSX (6.41 U mg^-1^ protein). Similarly, there were no changes in the activity towards the mannan substrates KGM (23.10 U mg^-1^ protein) and LBG (10.00 U mg^-1^ protein), and for barley 1,3-1,4-β-glucan (15.01 U mg^-1^ protein). Nguyen et. al. (7) reported that Fn3 linked to the endo-cellulase GH5 from goat rumen bacterium enhances the endo-cellulase activity on both soluble and insoluble cellulose substrates. Similarly, our results showed that *Pc*GH5 Fn3 increases cellulase activity towards CMC and Avicel but has no effect on xylanase, mannanase, and endo-1,3-1,4-β-glucanase activities.

Finally, we examined the role of CBM3 by comparing the activity of full-length *Pc*GH5 (*Pc*GH5CD-CBM11- Fn3-CBM3) with that of *Pc*GH5CD-CBM11-Fn3. As shown in Table 2, for cellulose substrates, the full-length enzyme showed a 1.11-fold increase in activity towards CMC (33.79 U mg^-1^ protein) and a 1.21-fold increase in activity towards Avicel (28.04 U mg^-1^ protein). For xylan substrates, the full-length enzyme showed a 1.22-fold increase in activity towards BWX (30.74 U mg^-1^ protein) and a 1.57-fold increase in activity towards OSX (10.09 U mg^-1^ protein). For the mannan substrates, the activities towards KGM (29.26 U mg^-1^ protein) and LBG (17.37 U mg^-1^ protein) were increased by 1.27-fold and 1.74-fold, respectively. Surprisingly, the full-length enzyme showed a 3.04-fold increase in activity towards barley 1,3-1,4-β-glucan (45.59 U mg^-1^ protein). These results suggest that barley 1,3-1,4-β-glucan is the most suitable substrate for full-length *Pc*GH5.

The presence of CBM3 in *Pc*GH5 plays an important role in substrate binding and catalytic efficiency: it significantly enhances activity towards various polysaccharide substrates, especially for 1,3-1,4-β-glucan and highly branched xylans and mannans (Table 2). Ran *et al*. [28] reported that when different substrates are used, the adsorption affinity of CBM3 from GH5 endo-cellulase III from *Trichoderma viride* for these substrate changes. CBM3 improves substrate targeting by positioning the catalytic domain closer to the polysaccharide surface, which increases the efficiency of hydrolysis [29]. The combination of CBM3, Fn3, and CBM11 in *Pc*GH5 has a beneficial effect on the substrates binding and enzymatic activity towards the β-1,4 glycosidic linkages of several polysaccharide substrates.

### FTIR Spectroscopy Analysis

**Fig. 5:**
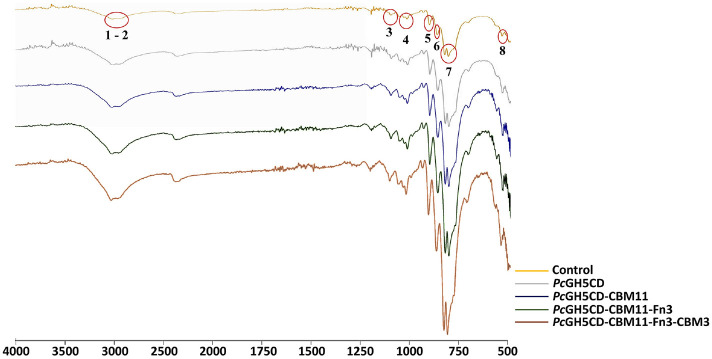
FTIR spectra of untreated and treated Avicel samples by of the full-length *Pc*GH5 enzyme and its truncated enzymes. Different colors represent the control, *Pc*GH5 and its truncated enzymes.

To confirm the contribution of each NCD (CBM11, Fn3, and CBM3) in full-length *Pc*GH5 to the hydrolysis of the substrate, FTIR spectroscopy was used to examine the chemical changes of the substrate structure after treated with full-length and truncated *Pc*GH5 enzymes, using untreated Avicel as a control. Avicel was chosen as the representative for FTIR spectroscopy analysis because it is a crystalline substrate in which the function of each NCD can be clearly seen, while other substrates cannot be used due to gelation during sample preparation. Compared with the control, the FTIR spectra of Avicel treated with all four enzymes showed significant peak shifts (Fig. 5), indicating significant changes in the main functional groups involved in the cellulose structure.

As shown in Table 4, a broad absorption band at 3,286 cm^-1^ is attributed to intermolecular hydrogen bonding, a critical factor influencing cellulose crystallinity and stability [30]. The peak observed at 2,918 cm^-1^ corresponds to the asymmetrical and symmetrical stretching of CH_2_ groups within the cellulose backbone, indicating the structural characteristics of the polymer [30]. The band at 1,162 cm^-1^ reflects C–O–C stretching associated with β- glucosidic linkages, providing evidence of intact glycosidic bonds [31]. Peaks at 1,018 cm^-1^ and 986 cm^-1^ denote C–O stretching and CO/ring stretching vibrations, respectively [30], while the peak at 983 cm^-1^ specifically indicates C–O stretching at the C–6 hydroxyl group [32]. The spectral region from 897 to 894 cm^-1^ corresponds to β-linkages in cellulose, confirming the structural adhesion of the polymer [33]. The peak at 667 cm^-1^ is attributed to out-of-plane bending of hydroxyl (OH) groups, further reflecting the presence of hydrogen bonding interactions within the cellulose matrix [31]. On the other hand, the peak appearing at 2,410 cm^-1^ is generally not related to cellulosic functional groups and may be due to impurities present in the sample.

As shown in Fig. 5, compared to untreated Avicel, *Pc*GH5CD showed significant degradation of Avicel as evidenced by the changes in FTIR spectra at wavenumbers 3286, 2918, 1162, 1018, 986, 983, 897-894, and 667 cm−1. Surprisingly, this result indicates that even without any CBMs or Fn3, the catalytic domain alone could hydrolyze the microcrystalline cellulose, Avicel. It is possible that the broken fragments on the surface structure of Avicel allow the *Pc*GH5CD enzyme to penetrate and break the hydrogen bonds between the cellulose chains in the crystalline regions, and then hydrolyzed the β-glucosidic linkages of the cellulose chains to produce sugars. Ludwiczek *et al*. [34] reported that in the absence of CBM, some exposed amino acids that can form hydrogen bonds, such as Asn, Ser and Thr, and hydrophobic interactions, such as Trp, which are located outside the active site, termed the secondary xylan-binding site of the single-domain of GH11 xylanase BcX from *Bacillus circulans*, can enhance the substrate specificity and catalytic activity of insoluble substrate. While Fatmawati *et al*. [2] found that conserved amino acid residues on the surface-exposed of the *Pc*GH5 catalytic domain structure, such as Trp4, Thr64, His70, and Asp250, which are located outside the active site, are important for the enzyme activity. Thus, these amino acid residues may be important in enabling *Pc*GH5CD to hydrolyze Avicel.

The addition of CBM11 resulted in more pronounced spectral shifts, particularly at peaks assigned to the intermolecular hydrogen bonding (3,286 cm^-1^), the CH_2_ asymmetrical and symmetrical stretching (2,918 cm^-1^), the COC at β-glucosidic linkage stretching (1,162 cm^-1^), the CO stretching (1,018 cm^-1^), the CO and ring stretching modes (986 cm^-1^), the C–O stretching at C–6 (983 cm^-1^), the β-linkages of cellulose (897-894 cm^-1^), and the OH out-of-plane bending (667 cm^-1^) (Fig. 5). These changes suggest that CBM11 supports the catalytic domain by increasing cellulose accessibility, which may facilitate the disruption of hydrogen bonds between the cellulose chains of crystalline cellulose and glycosidic linkages of cellulose chains.

The addition of Fn3 caused slight spectral shifts, characterized by decreased in the peaks assigned to the COC stretching at β-glucosidic linkages (1,162 cm^-1^), the C–O stretching at C–6 (983 cm^-1^), and the β-linkages of cellulose (897–894 cm-¹) (Fig. 5). These changes suggest that Fn3 likely contributes to the catalytic domain by increasing cellulose accessibility by modifying the surface of the Avicel structure. Similarly, Kataeva *et al*. [13] reported that Fn3 acts as a cellulose surface modifier to enhance the catalytic activity of cellulase towards crystalline celluloses. Although Fn3 has a limited effect on the spectral shift, its role in cellulose surface degradation is important, as the resulting surface modification may make cellulose fibers more accessible to the enzymatic activity of the catalytic domain.

The addition of CBM3 resulted in the most significant spectral shifts. As shown in Fig. 5, CBM3 exhibited FTIR spectra similar to CBM11, however, CBM3 showed more pronounced spectral shifts than CBM11 at all peaks. These results indicate the good efficiency of CBM3 in breaking intermolecular hydrogen bonds between cellulose chains in the crystalline regions of Avicel and its ability to facilitate the *Pc*GH5 catalytic domain enzyme in degrading Avicel.

The FTIR results were consistent with the specific activity results as shown in Table 2, where the enzyme activity of the *Pc*GH5 catalytic domain fused to the NCDs showed higher enzyme activity against Avicel compared to the *Pc*GH5 catalytic domain alone. These findings highlight the important role of Fn3 acting in the surface modification of Avicel, while CBM11 and CBM3 acted in enhancing the activity of *Pc*GH5CD by disruption the hydrogen bonds of crystalline cellulose and promoting the hydrolysis of glycosidic bonds of cellulose chains.

### Roles of Surface-Exposed Aromatic Residues in CBMs

To confirm the role of the key surface-exposed aromatic residues in the CBM3 and CBM11 that may be involved in substrate binding and enzyme activity, the exposed amino acid residues Trp17 in CBM11 and Trp51 in CBM3 of the full-length *Pc*GH5 enzyme were selected as representatives of CBM3 and CBM11, and then mutated individually by mutagenesis, yielding *Pc*GH5_CBM11_W17G and *Pc*GH5_CBM3_W51G, respectively. After cultivation, both mutant enzymes were purified using a HisTrap HP column, with both enzymes displaying a single band on SDS-PAGE (Fig. 1G).

As shown in Fig. 3E, compared to the control (without enzyme addition), *Pc*GH5_CBM11_W17G enzyme showed a significant loss of binding ability to all insoluble substrates, including barley 1,3-1,4-β-glucan, Avicel, BWX, OSX, KGM, and LBG, while the full-length wild-type *Pc*GH5 enzyme bound all polysaccharides except LBG, almost all of the enzyme bound to LBG (Fig. 3D). These results indicate that W17 in CBM11 plays a key role in binding all tested insoluble substrates. These results are consistent with previous report highlighting the role of aromatic amino acid residues of CBM11 in binding to insoluble substrates [25]. CBM11 from *Pc*GH5 could bind a wide range of insoluble substrates containing β-1,4 glycosidic bonds, a property that may contribute to the broad specific activity of *Pc*GH5.

Compared with the wild-type enzyme (Fig. 3D), *Pc*GH5_CBM3_W51G was able bind moderately to insoluble substrates, showing a significant loss of binding ability to barley 1,3-1,4-β-glucan, OSX, and LBG, while still binding to Avicel, BWX, and KGM (Fig. 3F). The CBM3 family has an extended planar binding surface composed of aromatic residues and has been proposed to interact via hydrophobic interactions with insoluble substrates [35]. These results indicate that W51 in CBM3 had greater specificity for binding to insoluble substrates compared to W17 in CBM11 of *Pc*GH5 enzyme.

The specific activities of the two mutant enzymes, *Pc*GH5_CBM11_W17G and *Pc*GH5_CBM3_W51G, were determined against various substrates (Table 5). The specific activity of *Pc*GH5_CBM11_W17G on CMC (27.01 U/mg protein), Avicel (23.05 U/mg protein), BWX (25.08 U/mg protein), OSX (4.50 U/mg protein), KGM (22.73 U/mg protein), LBG (4.98 U/mg protein), and barley 1,3-1,4-β-glucan (10.11 U/mg protein) were significantly lower than that of the full-length wild-type enzyme. Similarly, the specific activities of *Pc*GH5_CBM3_W51G on CMC (32.55 U/mg protein), Avicel (25.15 U/mg protein), BWX (28.88 U/mg protein), OSX (7.13 U/mg protein), KGM (26.44 U/mg protein), LBG (12.09 U/mg protein), and barley 1,3-1,4-β-glucan (15.59 U/mg protein) were also lower than that of the wild-type enzyme (Table 5). These results indicate that surface-exposed aromatic residues are important for the functions of *Pc*GH5 CBM11 and CBM3 in enzymatic activities.

Generally, CBMs support the catalytic domains in hydrolyzing insoluble substrate, however, some CBMs, such as CBM3 of the GH9 endo-cellulase (Cel9W) from *H. thermocellum* ATCC 27405, increase the enzyme activity on soluble substrate, CMC [36]. In our work, W51 in CBM3 had a slight effect on CMC, while W17 in CBM11 demonstrated the importance of the *Pc*GH5 catalytic domain enzyme activity on CMC (Table 5).

The specific activities of the two mutant enzymes towards Avicel, BWX, and KGM were not significantly different from those of the wild-type enzyme. On the other hand, for OSX, LBG, and barley 1,3-1,4-β-glucan, the specific activity of *Pc*GH5_CBM11_W17G was significantly lower than those of *Pc*GH5_CBM3_W51G and the wild-type enzymes, respectively (Table 5). This may be due to the fact that when W51 in CBM3 was replaced with glycine, CBM11 in *Pc*GH5 still functions to bind to all insoluble substrates, while *Pc*GH5_CBM3_W51G bound to Avicel, BWX, and KGM, but lost its ability to bind to barley 1,3-1,4-β-glucan, OSX, and LBG (Fig. 3F).

The specific activities of both mutant enzymes alone were lower than those of the wild-type enzyme in all substrates, especially for barley 1,3-1,4-β-glucan (Table 5). The specific activities of *Pc*GH5_CBM11_W17G and *Pc*GH5_CBM3_W51G enzymes were 10.11 U/mg protein and 15.59 U/mg protein, respectively, while the wild-type enzyme with both CBMs gave 45.59 U/mg protein, indicating a synergistic effect between the two CBMs in the wild-type *Pc*GH5 enzyme towards barley 1,3-1,4-β-glucan. These results may explain why *P. curdlanolyticus* B-6 produces two CBMs in a single protein.

### Mode of Action of Full-Length and Truncated *Pc*GH5 Enzymes on Polysaccharides

**Fig. 6:**
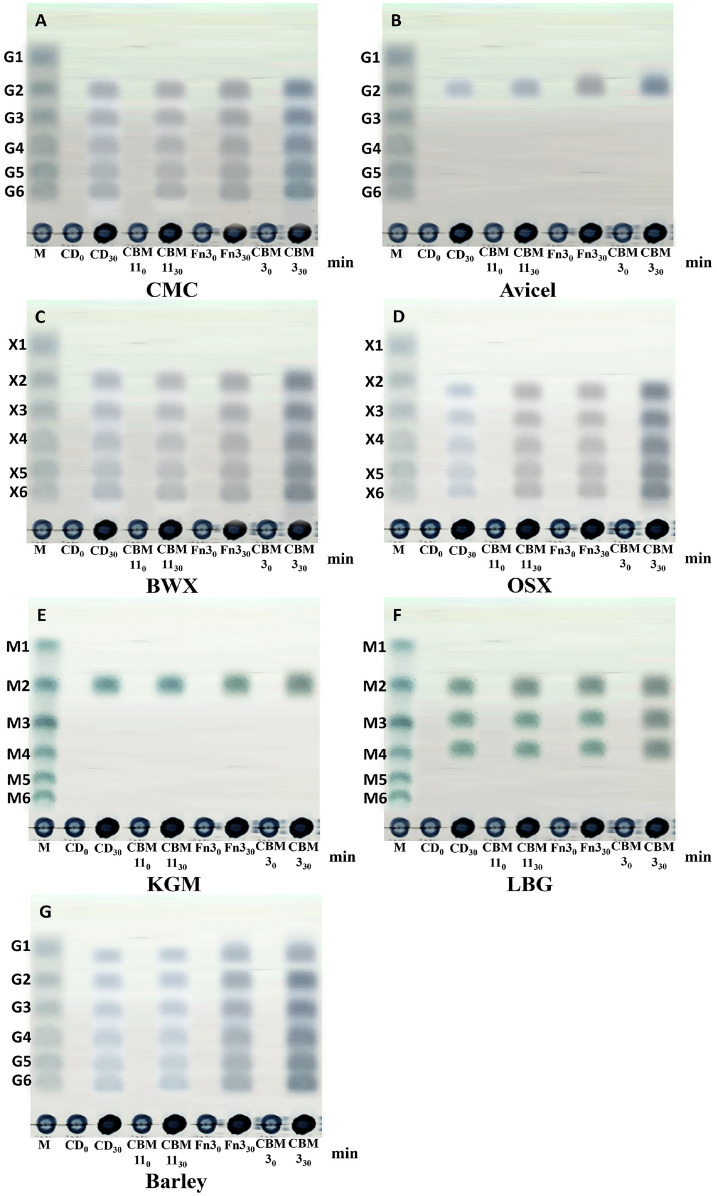
Thin layer chromatography (TLC) of hydrolysis products released from polysaccharides by purified *Pc*GH5CD, *Pc*GH5CD-CBM11, *Pc*GH5CD-CBM11-Fn3, and full-length *Pc*GH5CD-CBM11-Fn3-CBM3. The tested polysaccharides included carboxymethyl cellulose (CMC) (**A**), Avicel (**B**), birchwood xylan (BWX) (**C**), oat spelt xylan (OSX) (**D**), Konjac glucomannan (KGM) (**E**), locust bean gum (LBG) (**F**), and barley 1,3-1,4-β-glucan (**G**). Lane M represents size markers: glucose and cello-oligosaccharides (G1–G6), xylose and xylo-oligosaccharides (X1–X6), and mannose and manno-oligosaccharides (M1–M6). “0” and “30” represent the control and reaction at 30 min, respectively. Each polysaccharide (10.0 g ml^-1^) was incubated with enzymes (1 μmol l^-1^) in sodium acetate buffer (pH 6.0) at 50°C. For control, inactive enzymes were boiled for 15 min before use.

As shown in Fig. 6, *Pc*GH5CD, *Pc*GH5CD-CBM11, *Pc*GH5CD-CBM11-Fn3, and full-length *Pc*GH5 exhibited a similar mode of action, with similar product-release patterns from different polysaccharide substrates. However, the number of sugar spots on TLC of the released products tended to increase as the number of NCDs increased, a finding consistent with the binding between protein and polysaccharide (Fig. 3) and specific enzyme activities (Table 2). Thus, the presence of NCDs (CBM11, Fn3, and CBM3) in full-length *Pc*GH5 is likely to be important for substrate binding together with the increase in the enzyme activity towards the substrates.

Fig. 6 shows that the examined enzymes had similar hydrolysis product patterns for the β-1,4-linked polysaccharides. When combined with CMC, BWX, OSX, and barley 1,3-1,4-β-glucan, the enzymes produced a series of oligosaccharides ranging in length from dimers to hexamers (Fig. 6A, 6C, 6D and 6G), indicating an endo-acting mechanism. Based on the results, these enzymes cleave internal glycosidic bonds of the substrates, and NCDs do not alter the mode of action. On the other hand, the hydrolysis product of Avicel, which is microcrystalline cellulose, yielded only cellobiose (G2) (Fig. 6B). This may be due to the long and narrow substrate binding groove within the active site of the *Pc*GH5 catalytic domain. This conformation limits the ability of the complex Avicel structure, which consists of many cellulose chains tightly packed together with numerous hydrogen bonds, to enter the deep area of active site. Of note, this is a property of some endo-cellulases that hydrolyse crystalline cellulose [2].

Regarding the mannan substrates, full-length and truncated *Pc*GH5 degraded KGM, releasing only the dimeric product (Fig. 6E), which could be mannose-mannose or mannose-glucose [2]. For LBG, the enzymes produced tetrameric, trimeric, and dimeric oligosaccharides (Fig. 6F), which are composed of mannose-mannose and/or mannose-galactose linkages [2]. These results indicate that the hydrolysis products are substrate dependent. Phakeenuya *et al*. [20] reported that the type of substrate affects the cellulolytic and hemicellulolytic enzymes functions.

Cello-oligosaccharides, xylo-oligosaccharides, manno-oligosaccharides, and gluco-oligosaccharides produced by these enzymes have potential applications in various food and pharmaceutical industries due to their prebiotic and functional properties [2].

### The Feasibility of Applying NCDs in Enzyme Engineering and Biomass Degradation

Compared to other GH5 members, *Pc*GH5 is a highly diverse enzyme, containing endo-cellulase, endo-xylanase, endo-mannanase, and endo-1,3-1,4-β-glucanase. In addition, *Pc*GH5 exhibited significant hydrolysis of insoluble substrates (Table 2 and Fig. 6), which is due to the presence of three NCDs (CBM11, Fn3, and CBM3), which are more than the other NCDs connected to the other GH5 catalytic domains. These NCDs have been reported to have beneficial effects on substrate binding and enzymatic activity towards β-1,4 glycosidic linkages of several polysaccharide substrates [7, 12, 22, 29].

Currently, enzyme engineering by modifying the protein domain structure to enhance its catalytic activity in the hydrolysis of polysaccharides present in natural biomass is gaining attention. To improve the hydrolysis efficiency of the enzyme on target polysaccharides present in natural biomass, the three NCDs or the individual CBM11, Fn3, and CBM3 domains of *Pc*GH5 can be designed as chimeric proteins by adding them to the other GH5 catalytic domains or other GH enzyme families.

The chimera technique has been proven successful in improving the properties of enzymes. For example, after the incorporation of CBM11 from *R. thermocellum* celH (*Rt*CBM11) and the 1,3-1,4-β-glucanase from *B. subtilis* (BglS), the 1,3-1,4-β-glucanase activity was significantly increased, which is useful for the treatment of crude barley extract in the brewing industry [37]. Similarly, a chimera between the CBM11 of the cellulosomal enzyme *Ct*Lic26A-Cel5E from *C. thermocellum* and cellulase A from *D. turgidum* increased avicelase activity 1.74-fold [38].

A chimera between endopolygalacturonase I from *Chondrostereum* (*Stereum*) *purpureum* (EndoPG-I) and CBM3 from *H. thermocellum* endogalacturonase resulted in significantly increased catalytic efficiency in the saccharification of citrus pectin [39]. While CBM3c of GH9 endo-cellulase (Cel9W) from *H. thermocellum* ATCC 27405 is important for 1,3-1,4-β-glucanase and CMCase activities [10].

Although the precise function of the Fn3 domain remains uncertain, Fn3 linked to the endo-cellulase GH5 from goat rumen bacterium has been reported to enhance the endo-cellulase activity on both soluble and insoluble cellulose substrates [7]. Similarly, the Fn3-linked to GH3 β-glucosidase bglA from *Aspergillus niger* is important for the enzyme activity [40].

For useful industrial applications, the attractive properties of chimeric enzymes make them a good choice to improve the degradation of polysaccharides present in natural biomass. This presents an opportunity for new insights into protein domain modification to optimize enzyme properties and activity. Thus, *Pc*GH5 and its NCDs may be potential candidates for the bioconversion of economically important substrates such as natural biomass in the future.

## Conclusion

We have proposed distinct functions for the three NCDs, namely CBM11, Fn3, and CBM3, that are linked to the *Pc*GH5 catalytic domain. CBM11 is important in attacking crystalline cellulose (Avicel), low-branched and highly branched xylans and mannans, and 1,3-1,4-β-glucan. Fn3 acts only on cellulose. CBM3 significantly improves the ability of the catalytic domain to hydrolyse soluble and insoluble cellulose, low-branched and highly branched xylan and mannan, and 1,3-1,4-β-glucan. These three NCDs enhance the enzymatic activity of the catalytic domain on various polysaccharides as well as the hydrolysis of polysaccharides present in untreated natural biomass. Thus, we plan to study the role of these NCDs in the hydrolysis of natural biomass.

## Supplemental Materials

Supplementary data for this paper are available on-line only at http://jmb.or.kr.



## Figures and Tables

**Table 1:** The predictions of surface-exposed aromatic amino acids involved in hydrophobic interactions, number of surface-exposed aromatic amino acids to total amino acids, and percentage of surface-exposed aromatic amino acids in the protein (%) of each non-catalytic domain (NCD); CBM11, Fn3, and CBM3 from *Pc*GH5 compared to other characterized NCDs from various microorganisms.

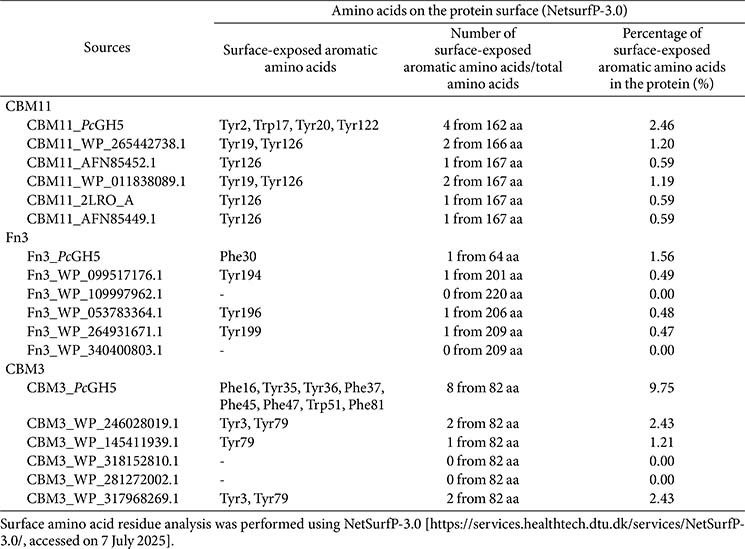

**Table 2:** Substrate specificity of the purified full-length *Pc*GH5 enzyme (*Pc*GH5CD-CBM11-Fn3-CBM3) and three truncated variant enzymes from *Paenibacillus curdlanolyticus* B6.

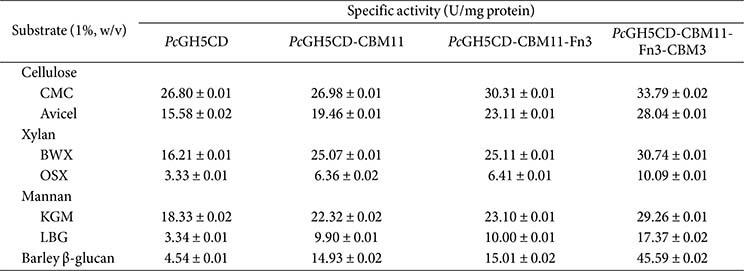

**Table 3:** Summary of molecular docking results of ligands G4, X4, and M4 interacting with amino acids located in the four binding subsites of the non-catalytic domains from *Pc*GH5 enzyme, namely CBM11, Fn3, and CBM3, respectively.

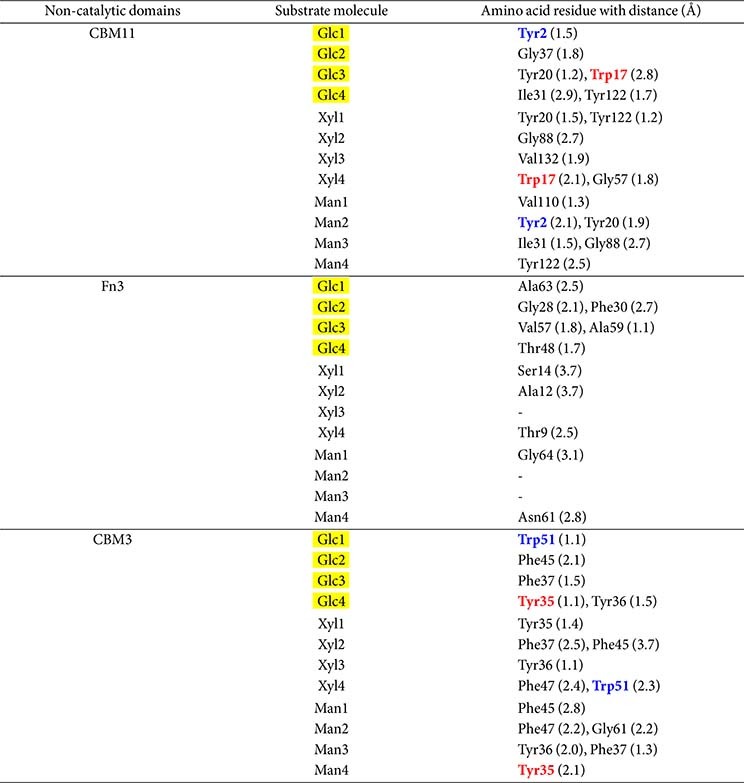

**Table 4:** Assignment and description of peaks corresponding to functional groups of Avicel by FTIR analysis.

Peak	Wavenumber (cm^-1^)	Corresponding components on Avicel	Reference
1	3286	Inter-molecular hydrogen bonding	[30]
2	2918	CH_2_ asymmetrical and symmetrical stretching	[30]
3	1162	COC at β-glucosidic linkage stretching	[31]
4	1018	CO stretching	[30]
5	986	CO and ring stretching modes	[30]
6	983	CO at C–6 stretching	[32]
7	897- 894	β-linkage of cellulose	[33]
8	667	OH out-of-plane bending	[31]

**Table 5:** Substrate specificity of the purified mutants *Pc*GH5_CBM11_W17G and *Pc*GH5_CBM3_W51G.