# Supplementary figures and images for: Role of the CBM11, Fn3, and CBM3 Domains in Enhancing the Multifunctional Enzymatic Activities of Glycoside Hydrolase Family 5 from Paenibacillus curdlanolyticus B-6
Source: J Microbiol Biotechnol. 2025 Oct 29;35:e2507030. doi: 10.4014/jmb.2507.07030 (PMC12603373; doi:10.4014/jmb.2507.07030)

Supplementary Figures

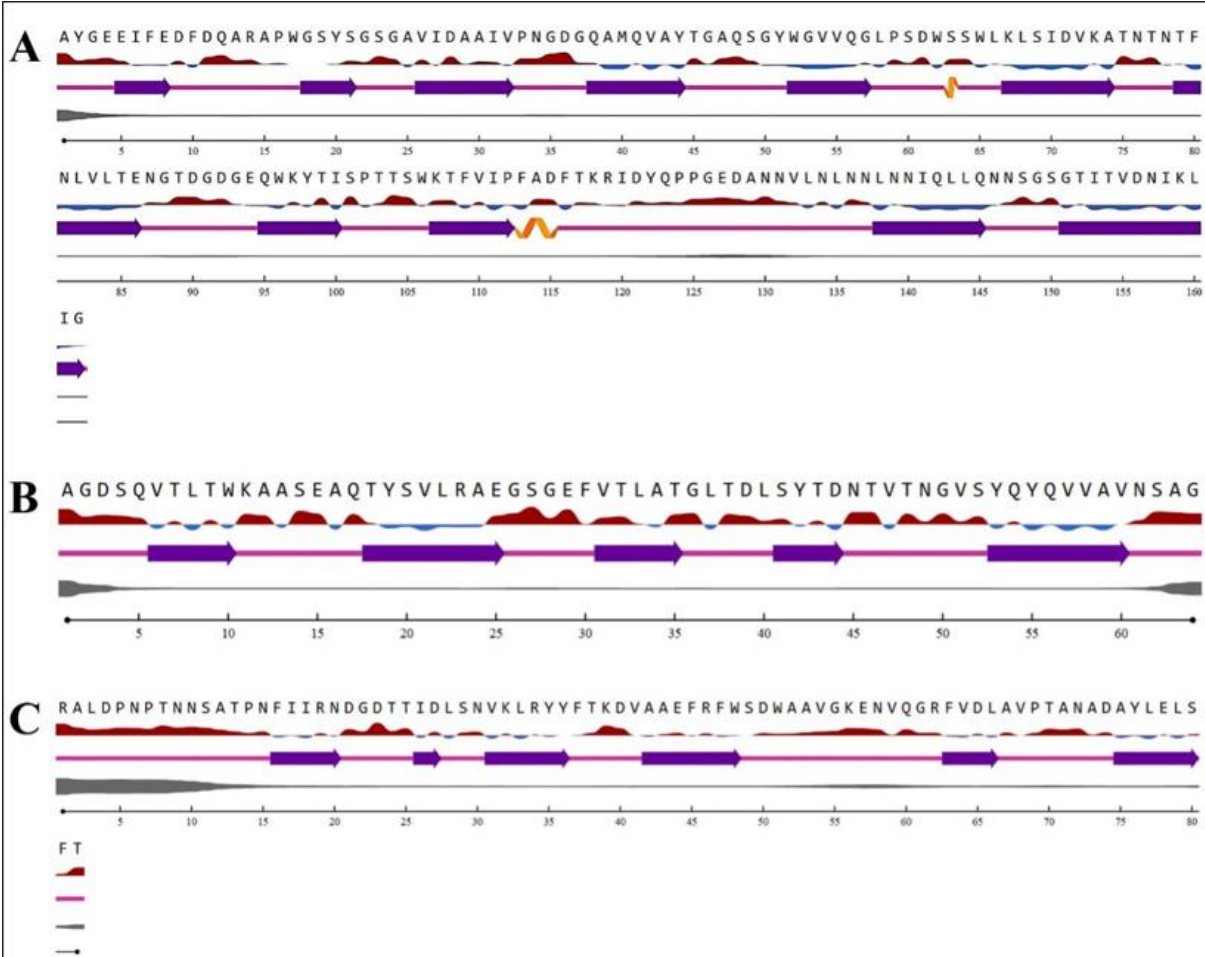

Fig. S1.

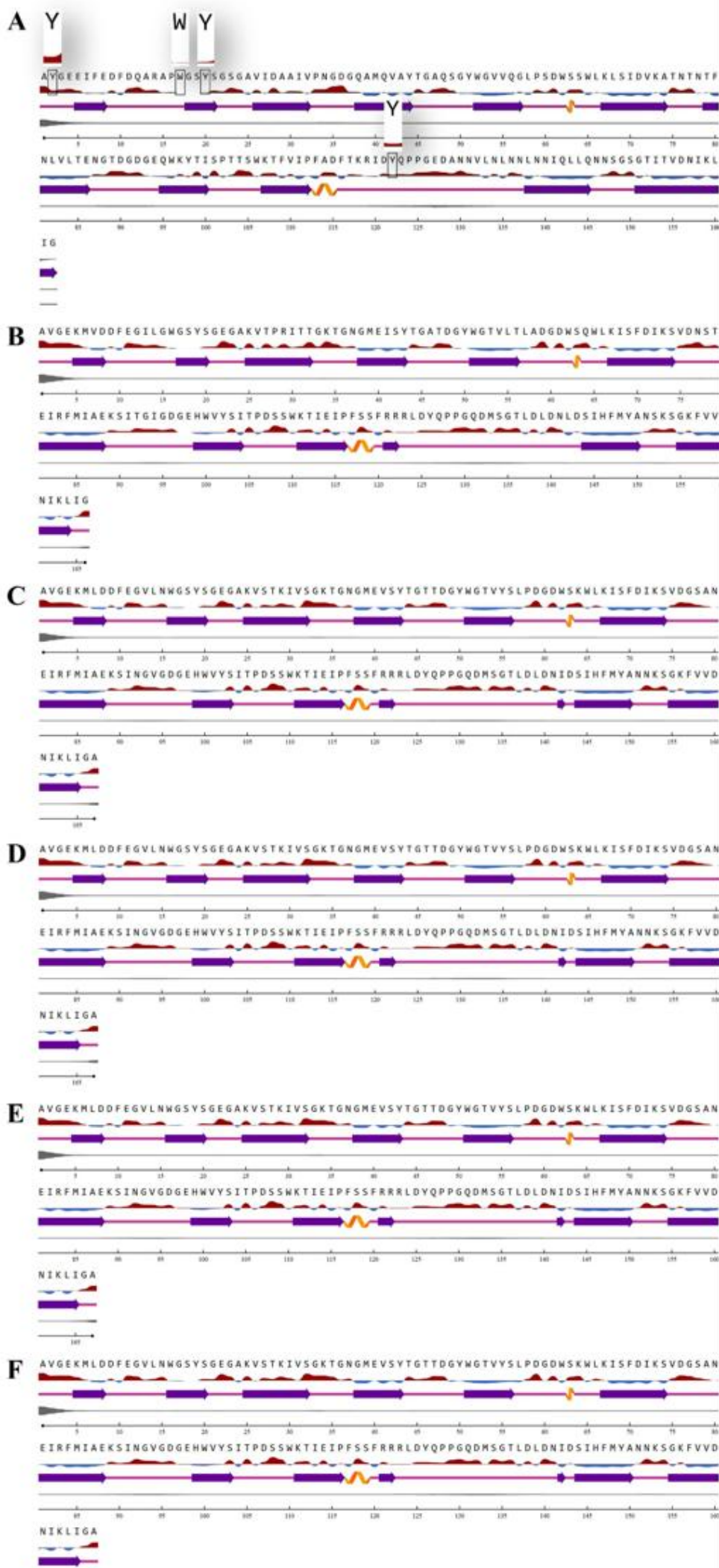

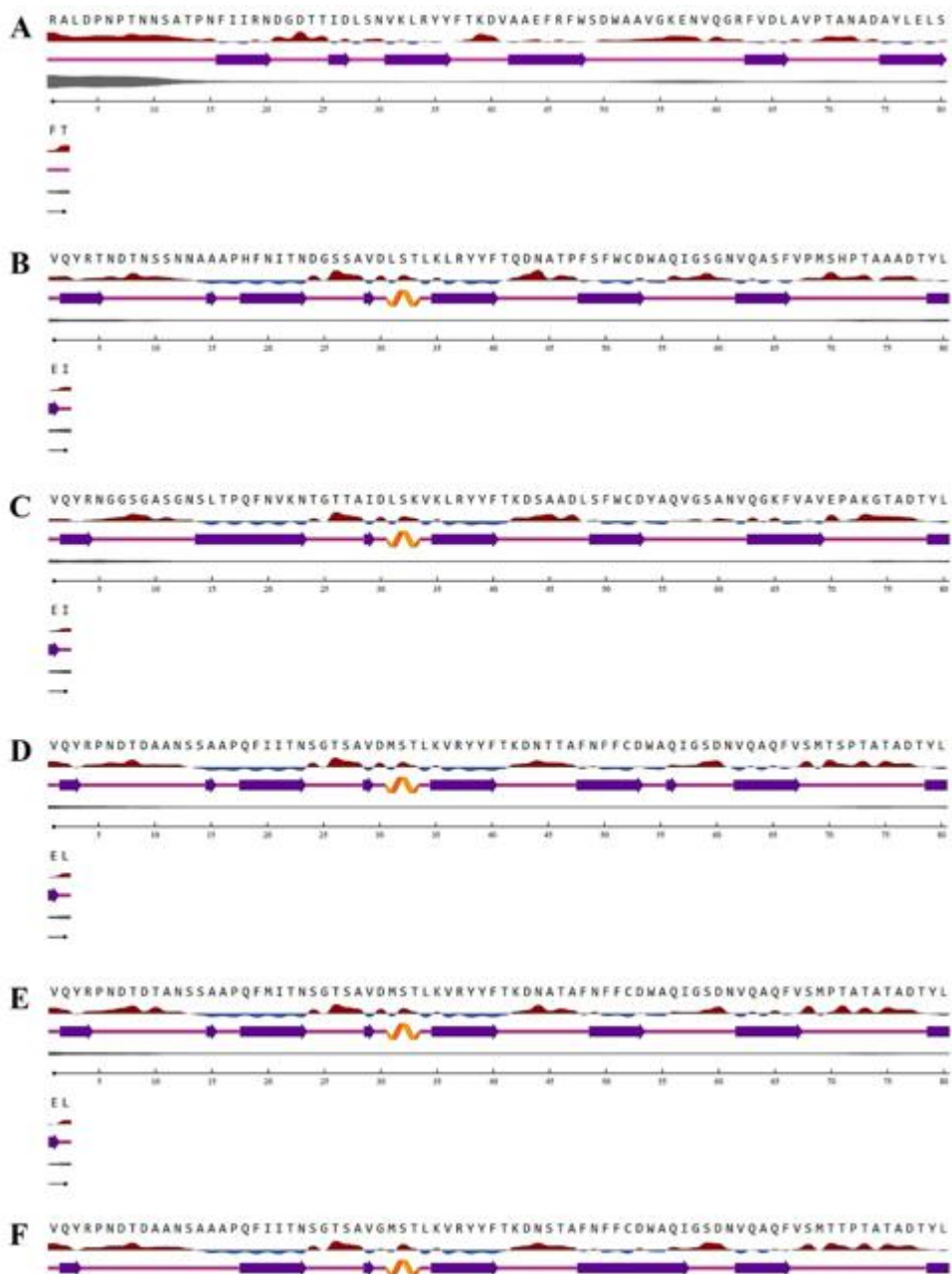

Fig. S3

Supplement: Supplementary file 1 [file jmb-35-e2507030-supple.pdf]
